# Transgenic Strategies for Sparse but Strong Expression of Genetically Encoded Voltage and Calcium Indicators

**DOI:** 10.3390/ijms18071461

**Published:** 2017-07-07

**Authors:** Chenchen Song, Quyen B. Do, Srdjan D. Antic, Thomas Knöpfel

**Affiliations:** 1 Laboratory for Neuronal Circuit Dynamics, Imperial College London, London W12 0NN, UK; song@knopfel-lab.net (C.S.); quyen.do-bao14@imperial.ac.uk (Q.B.D.); 2 Institute for Systems Genomics, Stem Cell Institute, UConn Health, Farmington, CT 06030-3401, USA; antic@uchc.edu; 3 Centre for Neurotechnology, Institute of Biomedical Engineering, Imperial College London, London SW7 2AZ, UK

**Keywords:** genetically encoded, voltage indicator, intersectional, transgenic, inducible expression, controlled recombination

## Abstract

Rapidly progressing development of optogenetic tools, particularly genetically encoded optical indicators, enables monitoring activities of neuronal circuits of identified cell populations in longitudinal in vivo studies. Recently developed advanced transgenic approaches achieve high levels of indicator expression. However, targeting non-sparse cell populations leads to dense expression patterns such that optical signals from neuronal processes cannot be allocated to individual neurons. This issue is particularly pertinent for the use of genetically encoded voltage indicators whose membrane-delimited signals arise largely from the neuropil where dendritic and axonal membranes of many cells intermingle. Here we address this need for sparse but strong expression of genetically encoded optical indicators using a titratable recombination-activated transgene transcription to achieve a Golgi staining-type indicator expression pattern in vivo. Using different transgenic strategies, we also illustrate that co-expression of genetically encoded voltage and calcium indicators can be achieved in vivo for studying neuronal circuit input–output relationships.

## 1. Introduction

To understand the dynamic interactions of neuronal circuits, covering large neuronal populations across multiple spatially distant brain regions, is a key focus in systems neuroscience. Recent progress in genetically encoded voltage and calcium indicators (GEVIs and GECIs respectively) now provides powerful tools toward this goal by allowing longitudinal monitoring from the same neuronal populations of defined cell classes [1,2,3,4,5]. GECIs (e.g., GCaMPs) have now been employed as a mainstream tool in systems neuroscience, yet calcium imaging provides only a surrogate and incomplete readout of neuronal activity. In contrast, by directly monitoring changes in membrane potential, GEVIs provide unparalleled opportunity to directly readout neuronal activity, including subthreshold fluctuations and hyperpolarisation.

Developments in the generation of Cre-dependent transgenic mice provides genetic approaches that achieve strong, cell type-specific expression of optical indicators in the mammalian brain, while bypassing the limitations of invasive gene expression techniques like electroporation and viral injections [6,7]. In combination with the use of the very strong tetracycline-responsive promoter element (TRE), modular intersectional transgenic strategies have been developed to achieve optimal cell class-specific indicator expression patterns [8].

GECIs can be targeted cytosolically to report changes in intracellular calcium concentrations, and since cell bodies contribute to a large fraction of the cytosol, GECI signals predominantly appear as “sparkling” cell bodies. GECI signals from dendrites and axons (“neuropil signals”) are often left unconsidered in densely labelled tissue, as they cannot be easily allocated to individual neurons. In contrast, GEVI expression needs to be strictly targeted to the plasma membrane to sense transmembrane voltage transients, and because membrane of the soma is only a small fraction of total membrane, the membrane-limited signals of GEVIs arise predominantly from dendritic and axonal membranes. For this reason, it is very difficult to attribute optical voltage signals from intermingled processes of many neurons to individual neurons. 

This problem could be resolved to facilitate single-cell resolution voltage imaging by targeting GEVIs sparsely to only a fraction of the neuronal population of interest. Conceptually, the idea of sparse cellular indicator expression bears resemblance to Golgi staining; a prime histological technique for resolving and segregating individual cells and their processes in a densely populated neuronal network. 

To achieve a strong cell class-specific but sparse expression of GECIs and GEVIs, we took advantage of the recently developed strategy to control Cre-dependent recombination via the use of a destabilized Cre variant, dCre [6]. This modified recombinase can be stabilized (that is, “de-destabilized”) using the antibiotic trimethoprim (TMP) that has no natural targets in mammals and penetrates the blood brain barrier [9]. Using a mouse line that expresses dCre under Rasgrf2A promoter [6], here we show that titratable recombination probability can be used to achieve strong intensity, but sparsely distributed (Golgi staining-like) indicator expression pattern in cortical layer II/III pyramidal neurons with either GCaMP6f (GECI) [10] or the voltage sensitive fluorescent protein (VSFP) VSFP Butterfly 1.2 (GEVI) [11,12].

The GEVIs used in the present study (from the Förster resonance energy transfer (FRET)-based VSFP Butterfly family containing mCitrine and mKate2 fluorescent protein pair) emit at two wavelengths. The signals in both wavelength bands can be spectrally deconvoluted from the GFP signals of GCaMP6f. We also show that, using these fluorescent proteins, the modular transgenic approach produced dual GEVI/GECI neuronal labelling, shown here in cortical layer II/III pyramidal neurons, which will allow monitoring of concurrent voltage and calcium activity in either the same neuron or in neighbouring neurons. 

## 2. Results

### 2.1. TMP Dose-Dependent Control of dCre-Mediated Recombination

**Figure 1:**
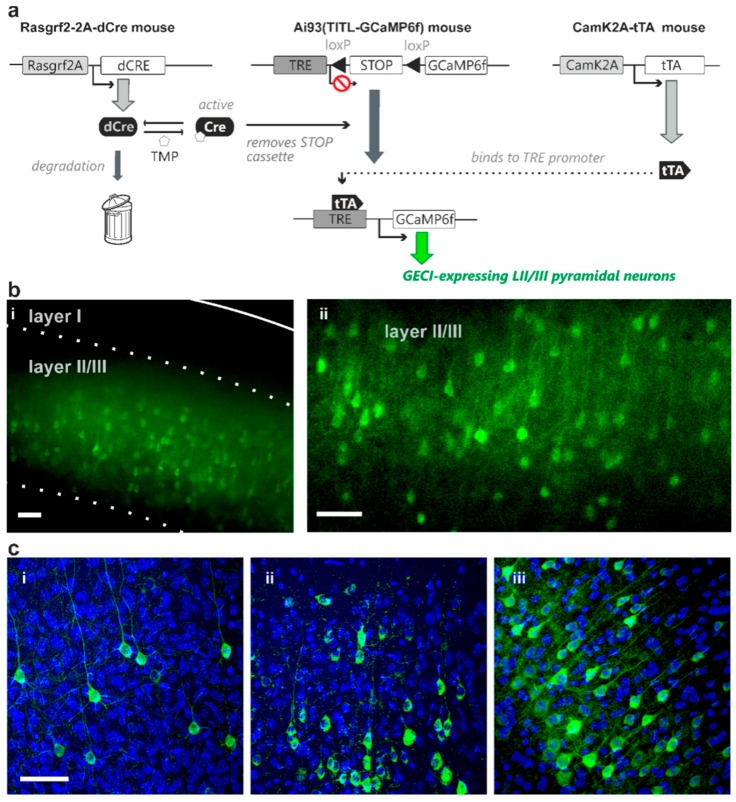
Representative images of adjusted GECI expression probability of layer II/III pyramidal neurons achieved via dose-dependent TMP administration. (**a**) Transgenic strategy using the combination of both Cre-recombinase and transactivator intersectionally controls indicator expression in identified cell populations in Ai93 GCaMP6f indicator transgenic mice. The transgenic strategy shown here uses a destabilized form of Cre-recombinase driven by Rasgrf2-2A promoter (Rasgrf2-2A-dCre) that can be “de-destabilized” using TMP to remove the STOP cassette in layer II/III neurons. Transactivator expression driven by CaMK2A promoter (CaMK2A-tTA) in pyramidal neurons is also required for binding to the TRE promoter in the indicator mice for indicator expression (GCaMP6f in this transgenic diagram). This combination of dCre and tTA facilitates adjustable indicator expression probability of layer II/III pyramidal neurons by titratable TMP administration (**b**) GCaMP6f expression density remained high with total TMP dose administered at 50 mg/kg body weight (**i**; imaged using wide-field epifluorescence microscopy; solid line marks the pia, dashed line marks cortical layer boundaries) and 0.5 mg/kg body weight (**ii**; using confocal microscopy, single plane) (**c**) GCaMP6f expression with total TMP dose administered at 0.005 mg/kg body weight (**i**), 0.03 mg/kg body weight (**ii**), and 750 mg/kg body weight (**iii**) TMP concentrations resulted in different levels of GCaMP6f expression density (confocal microscopy). DAPI nuclei-counterstaining shows, in addition to pyramidal neurons, dense cell population including glia, GABAergic interneurons and blood vessel-related cells. Scale bar = 50 µm; stack thickness = 15 µm.

We used a mouse line that carries a transgene cassette where dCre is under transcriptional control of the Rasgrf2 promoter that drives gene transcription in layer II/III neurons [6,13]. These mice were crossed with two mouse lines that carry (i) an expression cassette where the coding sequences for the GECI GCaMP6f is under transcriptional control of both Cre and tTA [8,14], and (ii) a cassette where expression of tTA is driven by the CaMK2A promoter [14]. In the presence of Cre-mediated recombination, these mice express GCaMP6f in layer II/III pyramidal cells [8]. In the absence of TMP treatment, dCre is degraded and Cre-mediated recombination prevented [9]. In the presence of increasing doses of TMP, increasing amounts of dCre are de-destabilised and are available to excise a STOP codon to permit tTA-activated transcription of the GCaMP6f gene (Figure 1a).

To test the control of indicator expression probability, animals were treated with different total doses of TMP ranging from 0.005 mg/kg body weight (single intraperitoneal injection) to 750 mg/kg body weight (three daily injections of 250 mg/kg for saturating expression levels). We initially treated animals with total TMP doses of 50, 5 and 0.5 mg/kg body weight (all single injections), and examined indicator expression after a two-week expression period. We observed comparable densities of expressing cells between 50 mg/kg and saturating 750 mg/kg body weight TMP administration (Figure 1(b-i); wide-field epifluorescence microscopy). Surprisingly, the lowest TMP dose used in this initial set of experiments (0.5 mg/kg body weight; Figure 1(b-ii); confocal microscopy) still induced indicator expression in the majority of the layer II/III pyramidal cell population (with the expected density of layer II/III pyramidal neurons derived from published CUX1 expression patterns [15]).

These initial results suggested that a TMP dose much lower than 0.5 mg/kg is needed to achieve desirable sparse Golgi-like indicator expression. We therefore administered total TMP dosages ranging from 0.005 to 0.05 mg/kg body weight (distributed over 1–2 injections per animal), and analysed the density of indicator expressing cells with confocal microscopy 1–3 weeks thereafter. In this set of experiments we also counterstained with DAPI, a fluorescent marker of cell nuclei. Notably, DAPI-stained nuclei include, in addition to those representing pyramidal cells, also those of GABAergic cells, glia cells and blood vessel-related cells. We therefore counted the indicator-expressing cells, normalized on the total count of nuclei and then scaled to the maximal density of layer II/III pyramidal cells that expressed the indicator under control of the stabilised Rasgrf2-dCRE gene product. We refer to this value as “scaled expression probability”. In this dose range, the fraction of layer II/III pyramidal cells that expressed the indicator increased with increasing total TMP dosage administered (Figure 1(c-i–iii), Table 1). 

The indicator expression levels did not depend on the fraction of expressing cells because recombination is “*all or not at all*” at the level of individual cells, while expression levels are controlled by the strong TRE promoter. We also explored expression durations longer than two weeks and TMP administration in older animals (aged 3–6 months) and did not find overt differences compared to our standard induction age of six weeks and two weeks of expression time at similar total TMP doses administered (data not shown). Similar TMP dose-dependent indicator expression was obtained with GEVI mice under the same transgenic configuration, where the Ai93 transgenic line was replaced by the Ai78 transgenic line such that Cre-mediated recombination leads to expression of the GEVI VSFP Butterfly 1.2, also in layer II/III pyramidal population. 

### 2.2. Sparse Expression of GEVI Uncovers the Morphologies of Individual Cells

**Figure 2:**
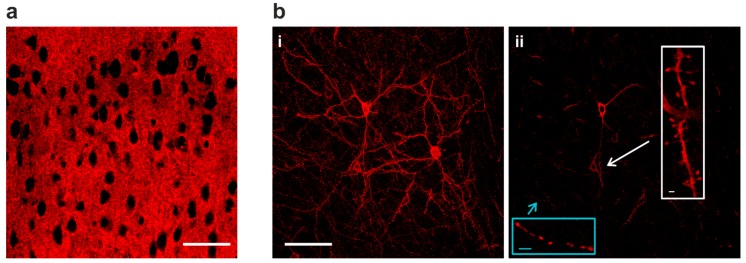
Sparse GEVI expression in vivo resolves subcellular structures. (**a**) At full expression density, GEVI optical signals are difficult to be assigned to individual neurons due to intermingled neuronal processes in dense populations. (**b**) Sparse Golgi staining-like GEVI expression density resolves single cells and subcellular morphologies (**i**) A 15 µm stack projection of three individual layer II/III pyramidal neurons. (**ii**) Single-plane image from the stack in (**b-i**). GEVI targeting achieves optimal membrane expression, and is capable of resolving subcellular structures in vivo, including axonal boutons (blue inset) and dendritic spines (white inset). Image scale bar = 50 µm; inset scale bar = 2 µm.

With VSFP Butterfly 1.2 or chimeric VSFP Butterfly expression in all cortical pyramidal cells, individual neurons could not be resolved due to intermingled neuronal processes (Figure 2a). Using the genetic approach described above (Figure 1a) along with a low dose of TMP for limited Cre-dependent recombination, strong Golgi staining-like GEVI expression was achieved (Figure 2(b-i)) and this sparse expression allowed resolving morphological details of individual cells. In single confocal planes, subcellular structures can also be clearly resolved, including axonal boutons and dendritic spines (Figure 2(b-ii)).

### 2.3. Modular Transgenic Strategies Allow Controlled Co-Expression of GECI and GEVI

**Figure 3:**
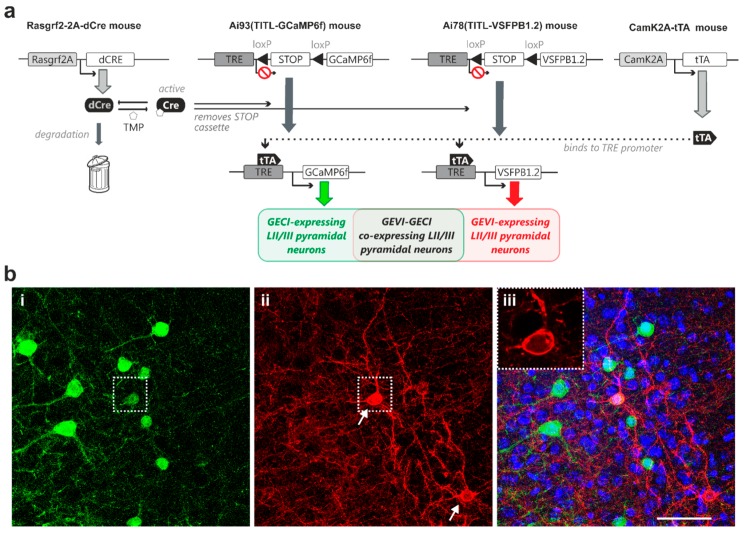
Dual-controlled co-expression of GECI (GCaMP6f) and GEVI (VSFP Butterfly 1.2) in somatosensory cortical layer II/III pyramidal neurons in vivo. (**a**) The combination of dCre and tTA allows for dual control of Ai78 VSFP Butterfly 1.2 and Ai93 GCaMP6f expression probabilities. This transgenic strategy resulted in GECI-expressing, GEVI-expressing and GECI/GEVI co-expressing populations. (**b-i**) Strong GCaMP6f fluorescence signal observed in perisomatic regions of individual pyramidal neurons. (**ii**) Strong VSFP Butterfly 1.2 fluorescence signal observed in the membranes of two pyramidal neurons (arrows). (**b-iii**) Merged GCaMP6f (green) and VSFP Butterfly 1.2 (red) fluorescence signals on DAPI nuclei counterstaining (blue) showing sparse Golgi staining-like indicator expression pattern. Inset: a single-plane confocal image of the indicator-expressing neuron, outlined by the dotted box in (**b-ii**), showing optimal GEVI membrane targeting. Note this cell co-expresses GCaMP6f and VSFP Butterfly 1.2. Scale bar = 50 µm.

Next, we expanded the above-described genetic approach to develop strategies for controlled expression of both a GECI and a GEVI in the same animal. To this end, we generated quadruple transgenic mice carrying both the GCaMP6f and the VSFP Butterfly 1.2 genes, and expression of both indicator proteins in layer II/III pyramidal neurons was under the control of inducible dCre and transactivator (Rasgrf2-dCre;CaMK2A-tTA;Ai78;Ai93, Figure 3a). Induction using titrated TMP dose resulted in observable sparse indicator expression, with subpopulations of layer II/III pyramidal neurons expressing either GCaMP6f (Figure 3(b-i)) or VSFP Butterfly 1.2 (Figure 3(b-ii)), and an overlapping sub-population co-expressing GCaMP6f and VSFP Butterfly 1.2 (Figure 3b; dotted box outlines an example neuron).

**Figure 4:**
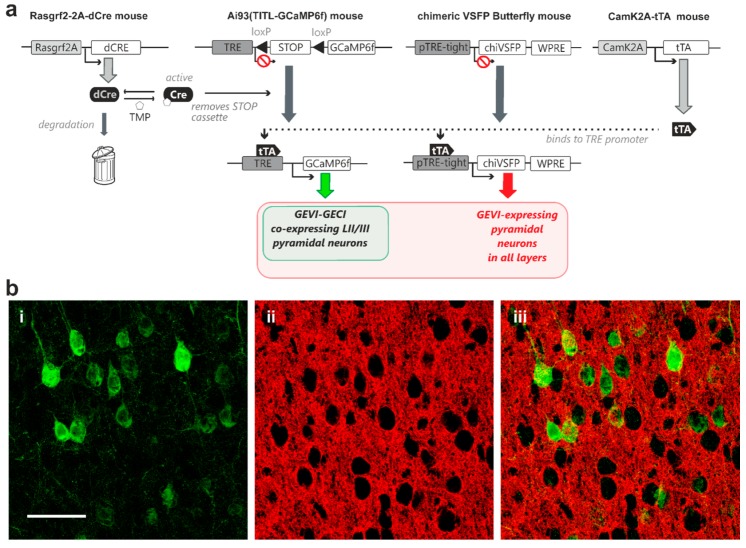
Expression of genetically encoded voltage indicator (chimeric VSFP Butterfly) in all cortical pyramidal neurons with controlled co-expression of genetically encoded calcium indicator (GCaMP6f) in layer II/III pyramidal neurons in vivo. (**a**) A transactivator, driven by CaMK2A promoter (CaMK2A-tTA) in pyramidal neurons, is required for binding to the TRE promoter for transcription activation in both chimeric VSFP Butterfly and Ai93 indicator mice. A STOP cassette removal using TMP-stabilized Cre-recombinase driven by Rasgrf2-2A promoter (Rasgrf2-2A-dCre) is only needed for the Ai93 line. This quadruple transgenic strategy therefore achieves full GEVI expression in all pyramidal neurons, with TMP dose-dependent adjustable GECI co-expression in layer II/III pyramidal neurons (**b-i**) Strong GCaMP6f fluorescence signal observed in perisomatic regions of individual pyramidal neurons (**b-ii**) A single confocal plane image showing strong chimeric VSFP Butterfly fluorescence signal from pyramidal neurons observed across all cortical layers (**b-iii**) Merged GCaMP6f (green) and chimeric VSFP Butterfly (red) fluorescence signals showing adjustable sparse GECI indicator expression over dense GEVI expression patterns. Scale bar = 50 µm.

A second quadruple transgenic combination was generated for controlled GCaMP6f expression in layer II/III pyramidal neurons in combination with expression of the GEVI chimeric VSFP Butterfly in all pyramidal neurons across all cortical layers. For this transgenic strategy, we crossed-in a line where chimeric VSFP Butterfly expression is under the exclusive control of the TRE element (Rasgrf2-dCre; CaMK2A-tTA; chiVSFP; Ai93; Figure 4a). Our TMP induction protocol achieved sparse GCaMP6f expression in layer II/III pyramidal neurons (Figure 4(b-i,ii)), while the chimeric VSFP Butterfly voltage indicator displayed a dense expression pattern (Figure 4(b-ii,iii)).

## 3. Discussion

Using a trimethoprim-inducible Cre-mediated activation of transcription under a strong TRE promoter, we attained controlled, sparse and strong Golgi staining-like expression of genetically encoded voltage and calcium indicators in vivo (Figure 1c). The expression is termed “strong” because the expression level in individual cells is high, and it is termed “sparse” because only a few cells express the fluorescent indicator. Sparse and strong GEVI expression allowed resolving morphologies of individual neurons and subcellular structures—including axonal boutons and dendritic spines—in the mammalian brain in vivo (Figure 2b-ii). By using different transgenic combinations, we also demonstrate that GEVI/GECI co-expression can be achieved in vivo, resulting in GEVI-expressing, GECI-expressing, and GEVI/GECI co-expressing populations in the same animal (Figure 3b-iii). Indicator-expressing neurons are a subpopulation within the population defined by the cell-class specific promotor, and we assume that recombination within the transgene does not affect the identity of the neuron or neuronal response properties [8,11,12]. 

### 3.1. GEVI and GECI Transgenic Approach

Until recently, in vivo application of GECIs and GEVIs was obtained using gene delivery techniques based on in utero electroporation or viruses [11,16,17,18,19,20]. Compared to these approaches, transgenic strategies achieve more reliable, reproducible, yet strong indicator expression patterns in the mammalian brain with minimal invasiveness [8,21], and allow for more stringent targeting of specific cell classes, as well as temporal and functional control of labelling [22]. In contrast to voltage sensitive dye (VSD) imaging and viral approaches that involve craniotomies for dye and gene delivery, the GEVI-based transgenic approach, when combined with thin-cranium mice, permits transcranial imaging through a fully intact skull for longitudinal studies of mammalian behaviour [4,8,11,23,24]. Moreover, the growing available palette of Cre driver lines and tetracycline transactivator system allows for intersectional transgenic approach for specific restricted expression patterns in vivo to achieve a reliable modular experimental strategy [6,25,26]. 

Rapid progress in the development of GEVIs and GECIs fuels the deciphering of neuronal circuit dynamics in systems neuroscience. GEVIs in particular permit the direct monitoring of cellular electrical activity, providing additional information on subthreshold activity, hyperpolarising events, and high frequency oscillatory activity at the population level of one specific cell type, and across spatially distributed populations of neurons [27,28]. This approach was explored in the past using classic VSDs injected into individual neurons [2,29]. The sparse GEVI expression protocol further advances the optical voltage monitoring approach by enabling cell class-specific voltage imaging at multiple single-cell resolution in vitro and in vivo.

### 3.2. Sparse Strong GEVI Expression Resolves Individual Cells and Subcellular Structures

The recent generation of GEVIs exhibit response dynamics compatible with recording fast action potentials (APs), as demonstrated in cell cultures [12,19,20,30,31]. However, resolution of APs from multiple neurons in vivo in mammalian brains is not yet routinely achieved due to the intermingled neuronal processes in dense cell populations that complicate precise optical signal allocation to individual cells.

The experimental strategies presented here offer a solution to this problem. Combination of the inducible dCre system with the intersectional transgenic approach for strong and cell class-specific indicator expression produces Golgi staining-like expression patterns with known cell identity (e.g., pyramidal neurons, Figure 1). Administration of a saturating TMP dose (750 mg/kg Figure 1c-iii) achieves a high density of indicator-expressing cells. We compared this density with expression pattern of CUX1 ([15], a DNA-binding protein homeodomain family that identifies layer II/III pyramidal neuron nuclei [32]), and we concluded that the Rasgrf2 promoter is active in most, if not all, layer II/III pyramidal neurons, hence when driving expression of stabilized dCre triggers Cre-mediated recombination in most, if not all, layer II/III pyramidal neurons. In contrast to the intermingled indicator expression at high recombination probability achieved by the saturating TMP dose (Figure 2a), strong but sparse expression pattern achieved by low-dose TMP administration resolves the morphology of distinct subcellular structures including axonal boutons and dendritic spines in single confocal planes in vivo (Figure 2b), opening the possibility for synaptic level functional monitoring in vivo. In our current study, a much lower dose of TMP is used than indicated in previous reports, to achieve limited recombination probabilities via the use of dCre [9]. A possible explanation could be the genomic location of the novel TIGRE locus—into which our indicator transgenes are inserted—is more Cre-accessible compared to the more widely used Rosa26 locus, thus lowering amounts of stabilized Cre required to achieve a similar recombination probability [8,22]. Although the titrated TMP induction protocol achieves adjustable recombination percentage amongst a cell population, the recombination event in any given cell is, in principle, “all-or-nothing”, thus the effective indicator expression (thereby signal intensity) remains strong in the expressing cells despite the low populational expression density (Figure 1, Figure 2 and Figure 3b). 

### 3.3. GEVI/GECI Co-Expression

GECIs like GCaMPs are currently employed as a mainstream tool in systems neuroscience as a surrogate indicator of neuronal activity via reporting changes in the intracellular calcium concentration of the expressing cell. Although GCaMPs offer large signal-to-noise ratio due to the substantial change in cytosolic calcium concentration upon activity, this optical signal has a mixed origin and, whilst useful for reporting suprathreshold AP events, is completely blind to synaptic inputs [2]. In contrast, GEVIs are ideally suited to monitor synaptic responses and input−output behaviour. A more powerful approach to full exploration of the circuit connectivity is to combine GEVIs with GECIs. Whilst this has been explored ex vivo using voltage and calcium sensitive dyes [33,34], such approaches remain impossible in vivo. 

In the present study, a successful dual GEVI/GECI neuronal labelling in vivo was achieved (Figure 3 and Figure 4) by using the modular nature of the intersectional transgenic strategies. In our transgenic animals, the fluorescence emission spectra from the mCitrine and mKate2 fluorescent protein pair in the FRET-based VSFP Butterfly family were sufficiently different from the GFP emission spectrum of GCaMP6f (Figure 3 and Figure 4), thereby providing the essential bases for future simultaneous optical imaging of both voltage and calcium activity of the same neurons in vivo. 

## 4. Materials and Methods

All animal experimental procedures were performed in accordance with the United Kingdom Animals (Scientific Procedures) Act 1986 under Home Office project and personal licences following appropriate ethical review. 

### 4.1. Animals 

Triple transgenic mice were generated to express GCaMP6f (Rasgrf2-dCre; CaMK2A-tTA;Ai93) or VSFP Butterfly 1.2 (Rasgrf2-dCre;CaMK2A-tTA;Ai78) in cortical layer II/III pyramidal neurons. Quadruple transgenic mice were generated to co-express VSFP Butterfly 1.2 and GCaMP6f in cortical layer II/III pyramidal neurons (Rasgrf2-dCre;CaMK2A-tTA;Ai78;Ai93), or to express chimeric VSFP Butterfly in all cortical pyramidal neurons and GCaMP6f in cortical layer II/III pyramidal neurons (Rasgrf2-dCre;CaMK2A-tTA;chiVSFP;Ai93). 

### 4.2. Inducible Control of Recombination Using Trimethoprim

Trimethoprim (Sigma, Germany) was dissolved in DMSO first at 100 mg/mL and then further serially diluted in DMSO. The TMP in DMSO solution was mixed 1:9 with 0.9% saline <1 min before intraperitoneal injection to avoid TMP precipitation. Triple or Quadruple transgenic mice received final TMP doses ranging from 0.005 to 750 mg/kg body weight and DMSO concentration was kept constant. 

### 4.3. Histology and Confocal Imaging

One to six weeks (see results) after TMP administration, mice were anaesthetized with pentobarbital sodium, and transcardially perfused with 4% paraformaldehyde, post-fixed overnight and cryoprotected with 25% sucrose. Coronal sections of 100 µm thickness were cut on a sledge microtome and stored in phosphate buffered saline. All sections were nuclei counterstained with DAPI and imaged under a Leica TCS SP5 confocal microscope, using 405 nm for DAPI excitation and 488 nm for GEVI/GECI excitation. Z-stacks of images were captured with 63× oil-immersion objective (Numerical Aperture = 1.4, 15 µm or 20 µm thick at 1.05 µm steps). Since GECI signals can be more accurately attributed to single cells, GCaMP6f-expressing sections were counted to determine TMP-indicator expression dose dependency. 

### 4.4. Ex Vivo Quantification of Indicator Expression

For GCaMP6f expression analysis, images were analysed post hoc using ImageJ Fiji 1.5 (NIH, MD, USA). Regions of interest (ROIs) corresponding to identified cells were manually defined. Straight line ROIs were placed across the two brightest points of the cytosolic region of selected cells. Mean fluorescence signal of these two brightest points (F_selected cell_) were normalised against the spatially averaged background signals (F_BG_) in the specific layers where the cells were selected. Selected cells are considered as expressing GCaMP6f if their F_selected cell_/F_BG_ ratio is equal to or greater than 1.5 and are considered as non-expressing otherwise. Only cell profiles that included a nucleus and were present in more than 3 z levels were counted. Quantification of DAPI-counterstained cell nuclei representing total number of cells present in each imaged region was manually performed using the ImageJ Cell Counter plugin. Quantitative data reported as mean ± SEM.

## Figures and Tables

**Table 1:** Titrated indicator expression probabilities.

(TMP) (mg/kg Body Weight)	No. of Animals	Total No. of Sections	Expressing Cells/All Nuclei	Scaled Expression Probability
750	5	25	25.29 ± 1.88%	1.00
0.03	5	25	20.02 ± 0.83%	0.79
0.005	2	10	15.68 ± 2.63%	0.62
0.001	2	4	4.51 ± 1.06%	0.18
